# Assessment of Smear Layer Removal and Push-Out Bond Strength Efficacy of Traditional and Herbal Root Canal Irrigants Across Different Root Levels: An In Vitro Study

**DOI:** 10.7759/cureus.64511

**Published:** 2024-07-14

**Authors:** Moksha Shah, Charles Patel, Kailash Attur, Nikunj Patel, Shylaja Attur, Manali Solanki, Aditi Patel, Palak Chhaya, Dhruvi Pandya, Hetal Maheshwari

**Affiliations:** 1 Department of Conservative Dentistry and Endodontics, Narsinhbhai Patel Dental College and Hospital, Sankalchand Patel University, Visnagar, IND; 2 Department of Oral Pathology, Narsinhbhai Patel Dental College and Hospital, Sankalchand Patel University, Visnagar, IND

**Keywords:** push-out bond strength, scanning electron microscopy (sem), smear layer evaluation, sodium hypoclorite, herbal irrigant, endodontic irrigant

## Abstract

Introduction: Endodontic therapy requires meticulous root canal debridement, pathogen elimination, and effective obturation to prevent microbial intrusion. The presence of the smear layer hinders sealer penetration, compromising sealing effectiveness. Sodium hypochlorite and chlorhexidine are esteemed endodontic irrigants. Herbal extracts like neem and tulsi, with antimicrobial and anti-inflammatory properties, show promise for root canal irrigation. The study aimed to evaluate the efficacy of various irrigants in removing the smear layer and enhancing push-out bond strength at different root canal levels.

Materials and methods: One hundred mandibular premolars with single canals were collected, and 50 samples each were used for the smear layer and push-out bond strength analysis. Neem and tulsi extracts were prepared for irrigation. Teeth were decoronated, and up to 30 (6%) canals were prepared and were randomly divided into five groups based on irrigants used. A smear layer examination was conducted after longitudinally sectioning the tooth and sections were observed in a scanning electron microscope (SEM). Obturation was done in the remaining samples, and the push-out bond strength was assessed using a universal test machine.

Results: Sodium hypochlorite showed the highest smear layer removal efficacy followed by chlorhexidine, neem, tulsi leaves with rose water extract, and normal saline. Chlorhexidine exhibited the highest push-out bond strength, with the coronal third presenting the strongest values, followed by neem, tulsi with rose water, normal saline, and sodium hypochlorite.

Conclusion: The study underscores the potential of herbal irrigants in endodontic therapy, indicating promising results while emphasizing the necessity for further clinical trials to validate their efficacy and other properties.

## Introduction

Successful endodontic therapy hinges on the meticulous debridement of the root canal system, elimination of pathogenic flora, and three-dimensional obturation with inert filling materials to impede microbial ingress from the oral environment and subsequent periapical dissemination [1].

The contemporary techniques used in root canal instrumentation form a finely granular and structureless layer that coats the inner root dentin, commonly known as the smear layer. It is composed of organic and inorganic substances that coat root canal surfaces, potentially harboring bacteria and impeding medicament and sealer penetration. It hinders effective obturation-material interaction, compromising sealing efficacy [2].

The existence of debris and smear layers has a detrimental impact on the adhesive properties of endodontic sealers to radicular dentin [3]. The preferred root canal irrigant should efficiently eliminate organic and inorganic smear layers from dentin without causing erosion and exhibit antibacterial properties. Sodium hypochlorite (NaOCl), at concentrations between 1% and 5.25%, is the primary irrigant in root canal therapy, known for its antimicrobial prowess and ability to dissolve tissues within the root canals [4].

Chlorhexidine gluconate (CHX), a bis-biguanide synthetic compound, efficiently eliminates *Enterococcus faecalis *at a 2% concentration and exhibits prolonged antimicrobial efficacy within the root canal due to its superior substantivity. As a final irrigating solution in endodontic procedures, CHX proves highly effective [2].

Herbal extracts, with antioxidant, antimicrobial, anti-inflammatory, and immune-enhancing properties, are gaining prominence as valuable irrigants in endodontic treatment. Indian neem, or *Azadirachta indica*, offers antibacterial leaf extract rich in compounds like nimbin and nimbidin. With diverse properties including antibacterial, antifungal, and anti-inflammatory effects, it shows promising results for root canal irrigation [5].

*Ocimum sanctum* L., commonly known as "tulsi" in Hindi and "holy basil" in English, is a widely used medicinal plant in Indian households for addressing diverse health concerns. The essential oil extracted from its leaves contains eugenol, a phenolic compound associated with antimicrobial properties [6]. The smear layer removal effect of endodontic irrigants can be assessed by scanning electron microscopic (SEM) examination along the inner wall of root dentin. Restoration durability hinges on the bond strength, especially between dentin and root-end fillings and smear layer removal capacity of root canal irrigants, crucial for root canal treatment success. Evaluation often employs the push-out bond strength test, deemed as the most reliable for assessing material adhesion to surrounding dentin [7].

Hence, the study aimed to assess the effectiveness of saline, neem, chlorhexidine, tulsi leaves with rose water extract, and sodium hypochlorite irrigants in removing the smear layer from different levels of the root canal (coronal, middle, and apical thirds) and evaluate the push-out bond strength of the obturating material at corresponding thirds of the root. The objectives of this study are as follows: 1) to assess the effectiveness of various irrigants (i.e., saline, neem, chlorhexidine, tulsi leaves with rose water extract, and sodium hypochlorite) in removing the smear layer from the coronal, middle, and apical thirds of the root canal; 2) to evaluate the push-out bond strength of the obturating material at the coronal, middle, and apical thirds of the root canal after irrigation with the specified solutions.

The null hypothesis was that there is no significant difference in smear layer removing efficiency and push-out bond strength among saline, neem, chlorhexidine, tulsi leaves with rose water extract, and sodium hypochlorite at each respective third of the root.

## Materials and methods

Hundred extracted human single-rooted mandibular premolars with single canals were collected from the Department of Oral and Maxillofacial Surgery. Fifty samples were used for the smear layer and push-out bond strength examination each. Radiographs were taken to ensure a single canal and the absence of resorption, cracks, fractures, or calcifications. Ultrasonic scaling was done to remove calculus and soft tissues, and the teeth were stored in a 0.1% thymol solution.

Preparation of neem extract

Mature neem leaves were collected, weighing 25 grams, and were subsequently cleaned with sterilized distilled water. These leaves were macerated and then mixed with 50 mL of 100% ethanol for one to two minutes. The resulting mixture was filtered using Whatman filter paper, and the prepared extract was collected. Next, the mixture was placed in a water bath heated to 45-50 °C for one hour. Finally, the prepared irrigating solution was stored in an airtight container.

Preparation of tulsi leaves with rose water extract

One hundred grams of tulsi leaves were gathered and washed thoroughly with sterilized distilled water. Rose water was then added to the washed leaves, and the mixture underwent fermentation. Subsequently, the mixture underwent distillation at 100°C, and the obtained solution was used for irrigation.

Sample preparation and irrigation protocols

To achieve a standard length of 15 mm, the teeth underwent decoronation at the cementoenamel junction using low-speed diamond discs attached to a contra-angled handpiece. Copious water irrigation was done throughout the procedure to prevent overheating. Fifty samples were randomly divided into five groups (n = 10) each: Group 1 (normal saline), Group 2 (neem extract), Group 3 (chlorhexidine), Group 4 (tulsi leaves extract with rose water), and Group 5 (sodium hypochlorite).

Access opening was done, the working length was determined by introducing 15 K-file into the canal, and each canal was prepared till the working length with the Neoendo S NiTi rotary system up to size 30 (6%) taper. The canals were then irrigated between each instrument change to avoid unwanted changes to the root canal dentin.

To achieve a uniform distribution of solutions, the roots were irrigated using a conventional irrigation syringe and a 27 gauge side-vented needle, employing an apical-coronal motion within 1 mm of the working length. All the specimens were irrigated with their respective irrigating solution. Subsequently, roots were thoroughly rinsed with 5 mL of sterilized distilled water for one minute to eliminate the respective irrigating solution. After that, 50 samples were obturated with 30 (6%) cones using AH plus as a sealer for the push-out bond strength test.

SEM examination

**Figure 1:**
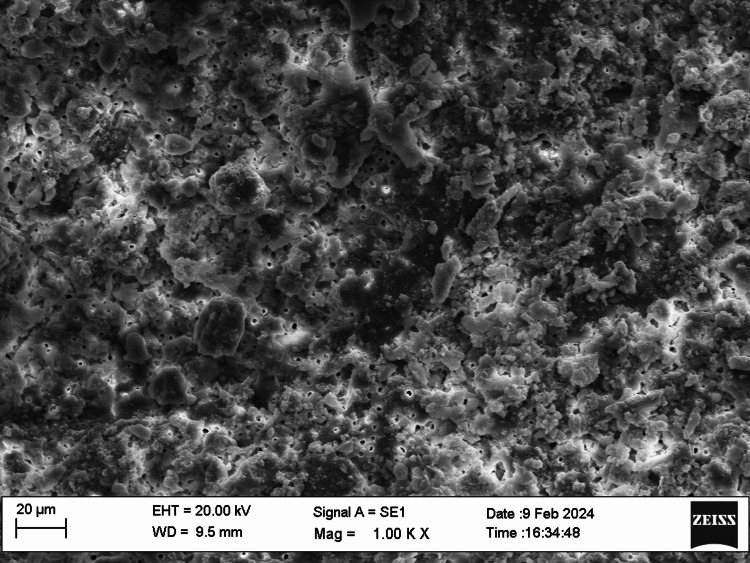
Smear layer removal (score 1)

**Figure 2:**
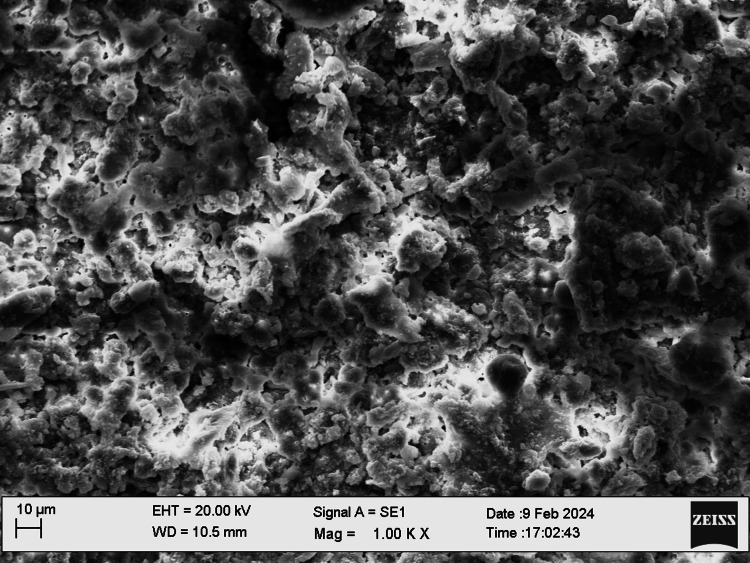
Smear layer removal (score 2)

**Figure 3:**
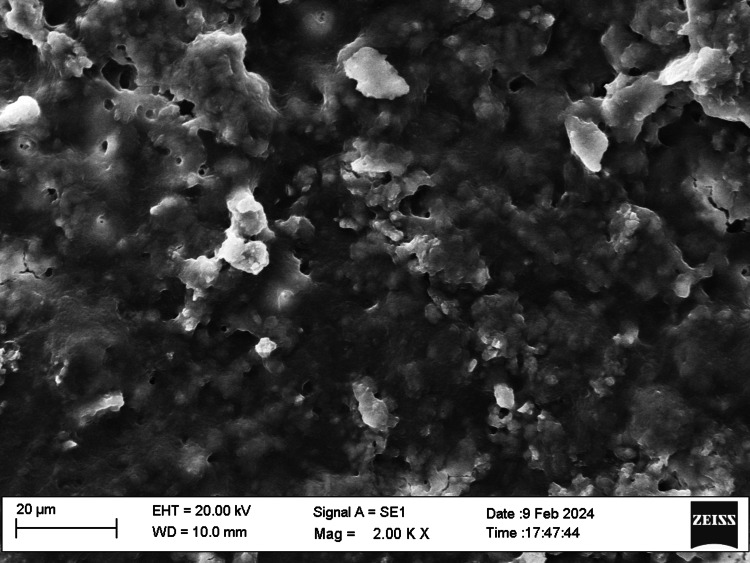
Smear layer removal (score 3)

**Figure 4:**
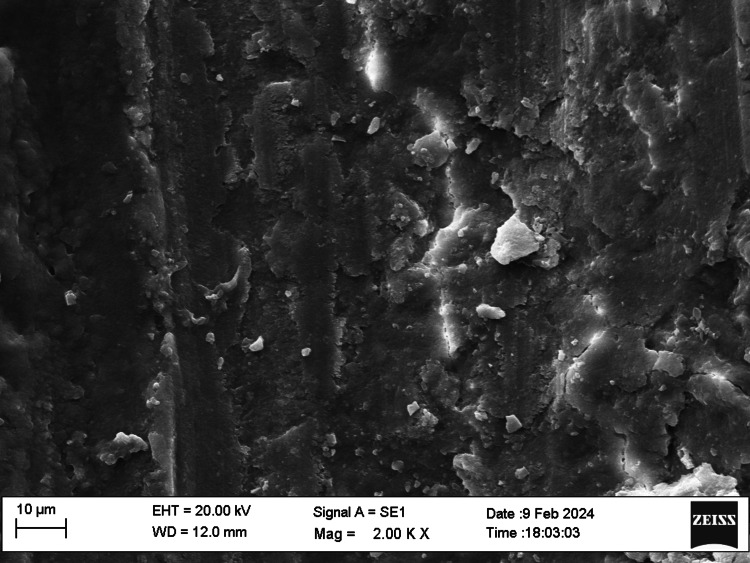
Smear layer removal (score 4)

**Figure 5:**
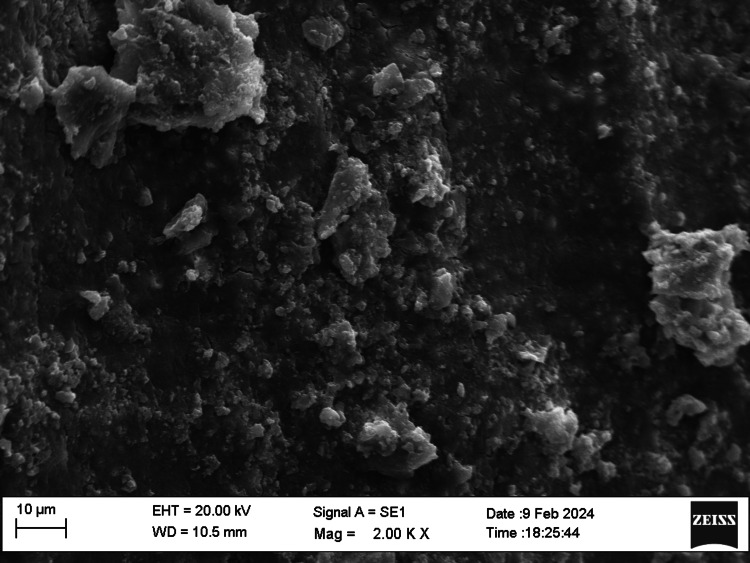
Smear layer removal (score 5)

Fifty root canal samples were dried with paper points and were longitudinally grooved with diamond discs, sectioned, then fixed in glutaraldehyde, dehydrated, and dried. Specimens were labeled, mounted, gold-sputtered, and observed via SEM at 1000 to 2000x magnification at apical, middle, and coronal thirds. Photographs were taken and were evaluated by two blinded examiners. The smear layer scoring criteria were as follows: score 1, no smear layer; score 2, <25% smear layer; score 3, >50% uneven smear layer; score 4, thin, homogenous smear layer; and score 5, thick, nonhomogeneous smear layer. Data were statistically analyzed using IBM SPSS Statistics, version 20.0 (released 2011, IBM Corp., Armonk, NY), with a significance level of p ≤ 0.05. The chi-square test was used for assessing the smear layer, while the push-out bond strength examination utilized repeated-measure ANOVA tests for analysis. The scoring criteria are shown in Figures 1-5.

Push-out bond strength examination

**Figure 6:**
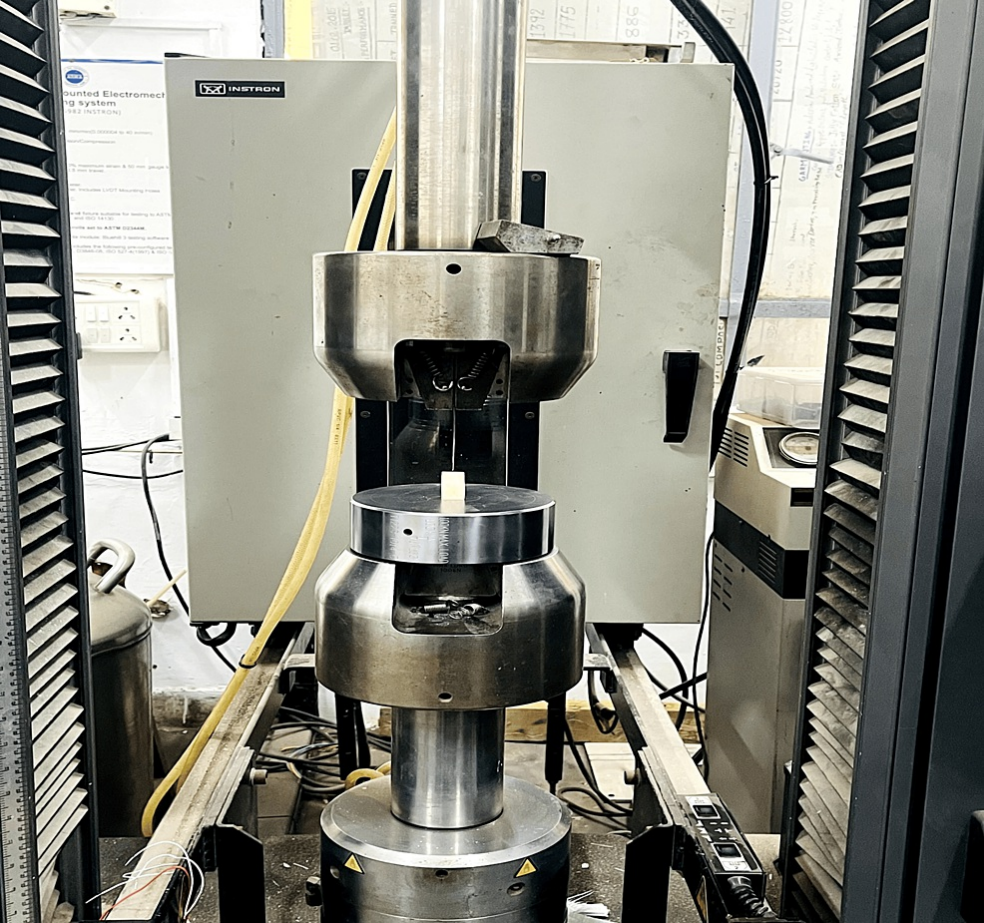
Universal testing machine

Following incubation under controlled conditions (37°C, 100% humidity) for one week to facilitate sealer setting, obturated samples underwent precise perpendicular sectioning along their longitudinal axis with a diamond disc. Three 2 mm-thick segments of 3, 7, and 11 mm from the apex, corresponding to the apical, middle, and coronal regions, respectively, were obtained. The samples were mounted onto acrylic resin blocks and were subjected to a push-out test using a universal testing machine (Instron, USA) equipped with segment-specific plungers, as shown in Figure 6. The load was applied at a controlled rate of 1 mm/min. The plunger was engaged with the root filling only during the loading process. Bond failure was identified by a sudden load decline, and displacement values were recorded in MPa (megapascals) ensuring precise and methodical assessment of root filling retention.

## Results

Table 1 shows the mean and standard deviation of scores achieved for the smear layer removal across all three root regions while using different root canal irrigants. Significant differences were found between the coronal and apical thirds in the smear layer removal efficacy, while it was insignificant between the coronal and middle thirds. Overall, the irrigants displayed reduced efficacy in the apical third across all groups. The hierarchical order of effectiveness observed across the entirety of the root canal was as follows: sodium hypochlorite exhibited the highest efficacy, followed by chlorhexidine, neem extract, tulsi leaves with rose water extract, and normal saline.

**Table 1:** Smear layer scores at various root levels while using different root canal irrigants Level of significance: p ≤ 0.05, * significant, ** non-significant

Group	Root portion	n	Maximum score	Minimum score	Mean	SD	p-value
Normal saline	Coronal	10	5	4	4.6	0.51	≤0.001*
	Middle	10	5	4	4.7	0.48	
	Apical	10	5	4	4.8	0.42	
Neem	Coronal	10	2	1	1.7	0.48	≤0.001*
	Middle	10	3	2	2.2	0.42	
	Apical	10	3	2	2.4	0.52	
Chlorhexidine	Coronal	10	2	1	1.2	0.42	≤0.001*
	Middle	10	2	1	1.5	0.52	
	Apical	10	2	1	1.6	0.52	
Tulsi leaves with rose water extract	Coronal	10	3	2	2.6	0.51	≤0.001*
	Middle	10	4	2	2.9	0.56	
	Apical	10	4	3	3.5	0.53	
Sodium hypochlorite	Coronal	10	1	1	1	0	≤0.001*
	Middle	10	1	1	1	0	
	Apical	10	2	1	1.4	0.51	

**Figure 7:**
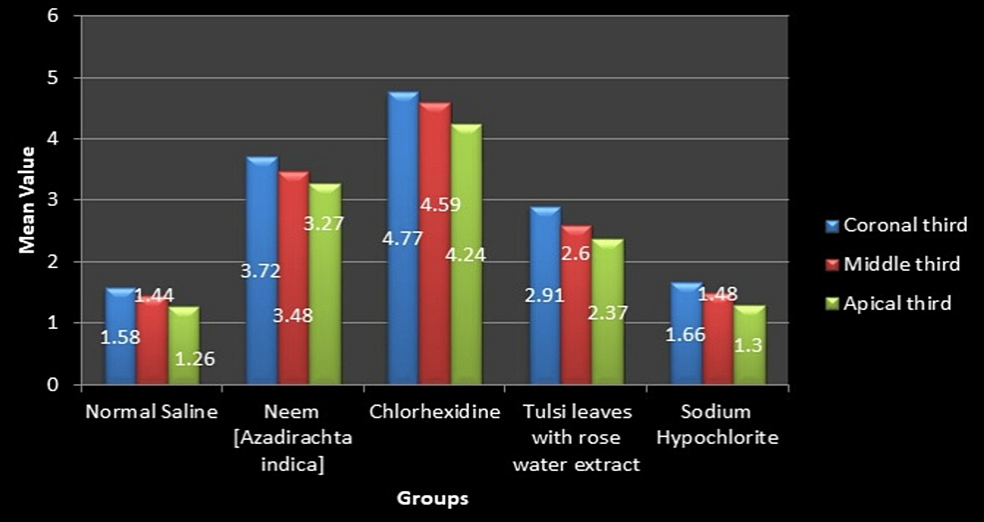
Mean push-out bond strength (Mpa) among various root levels

Table 2 shows that the mean push-out bond strength (Mpa) of the chlorhexidine group exhibited the highest values across all root portions, with the coronal third showing the highest strength (4.77 ± 0.17), followed by the middle third (4.59 ± 0.08) and apical third (4.24 ± 0.11). This was followed by the neem group, tulsi leaves with rose water extract group, normal saline group, and sodium hypochlorite group. A statistically significant difference in the push-out bond strength was observed along the root levels in all groups. Figure 7 shows the graph of the mean push-out bond strength of all the groups at different root levels.

**Table 2:** Push-out bond strength at different root levels Level of significance: p ≤ 0.05, * significant, ** non-significant

Groups	Root portion	Number	VAS		p-value
			Mean	SD	
Normal saline	Coronal	10	1.58	0.13	≤0.001*
	Middle	10	1.44	0.14	
	Apical	10	1.26	0.12	
Neem (*Azadirachta indica*)	Coronal	10	3.72	0.14	≤0.001*
	Middle	10	3.48	0.13	
	Apical	10	3.27	0.13	
Chlorhexidine	Coronal	10	4.77	0.17	≤0.001*
	Middle	10	4.59	0.08	
	Apical	10	4.24	0.11	
Tulsi leaves with rose water extract	Coronal	10	2.91	0.15	≤0.001*
	Middle	10	2.60	0.12	
	Apical	10	2.37	0.14	
Sodium hypochlorite	Coronal	10	1.66	0.08	≤0.001*
	Middle	10	1.48	0.10	
	Apical	10	1.30	0.16	

## Discussion

Herbal extracts offer advantages, such as cost-effectiveness, accessibility, extended shelf life, low toxicity, lack of microbial resistance, improved patient tolerance, and biocompatibility when used for endodontic purposes [8].

Single-rooted mandibular premolars were selected for their frequent use in orthodontic extractions, facilitating accessibility. To facilitate thorough cleaning and optimal penetration of the irrigating solution, the biomechanical preparation was done up to 30 (6%) files with the crown-down technique.

Syringe-based irrigation offers the ability to regulate the volume of solution dispensed and also the depth of penetration into the apical third of the canal. Consequently, for enhanced safety measures, it is recommended to utilize syringes ranging from 1 to 5 milliliters in capacity [9]. Findings align with studies by Charlie et al. [10], showing effective cleaning in the middle and coronal thirds, with better flow in larger canal diameters. Syringe irrigation with a 3 mL syringe and a side-vented 27-gauge needle tip was preferred in our study.

SEM allowed examination of root canal surface morphology at magnifications from ×50 to ×5000, enabling observation of debris fragments of varying sizes. Lower magnifications visualized larger debris fragments, while higher magnifications aided in identifying the smear layer and dentinal tubule openings. SEM at 1000× to 2000× magnification assessed the smear layer at the coronal, middle, and apical regions of the root canals.

In the present study, a statistically significant difference was observed in the mean removal of the smear layer from the root canal dentin when comparing normal saline, neem extract, chlorhexidine, tulsi leaves with rose water extract, and sodium hypochlorite irrigating solutions. The observed order of effectiveness across the entire root canal was as follows: sodium hypochlorite > chlorhexidine > neem extract > tulsi leaves with rose water extract > normal saline.

Root canal fillings serve to prevent leakage from the oral cavity and peri-radicular tissue. Sealer binds filling material, maintaining a void-free, compact mass adhered to the canal wall, while gutta-percha fills the radicular space. Bond strength testing is commonly employed to evaluate the efficacy of the bond between root-filling materials and radicular dentin. In our study, it was found that the decreased bond strength from the coronal to the apical direction indicated diminished binding capacity of root canal sealants in this orientation. The above findings were similar to those of the study conducted by Khanvilkar et al. [10].

NaOCl is widely known for its lubricating and antibacterial characteristics, facilitating organic matter dissolution and smear layer removal from the root canal. It may lead to the degradation of root dentin collagen, impacting the integrity of essential pyridinoline crosslinks crucial for epoxy resin sealer adherence, and therefore it exhibited the least push-out bond strength in the present study.

Chlorhexidine, widely used for root canal irrigation, is recommended at concentrations of 0.1-0.2% and 2%, respectively. Combining 2.0% chlorhexidine gluconate with 17% EDTA proved effective in dentin wall cleaning, suggesting its potential as an alternative irrigant [2]. CHX, with its surface-active action, enhances dentin surface energy for improved wettability, crucial for resin sealer adherence. It also augments dentin substrate cationic charge, promoting bonding with AH Plus sealer, particularly to the organic phase of radicular dentin [11]. In our study, chlorhexidine demonstrated the best push-out strength.

This study has shown the successful and proficient utilization of an irrigant capable of eliminating the smear layer, facilitating the disinfection of dentinal tubules, and promoting the push-out bond strength.

Herbal endodontic irrigants have potent antimicrobial, anti-inflammatory, antiseptic, and antioxidant properties; are biocompatible, accessible, and cost-effective; and have low toxicity, minimal tooth staining, and reduced *Enterococcus faecalis *resistance [12].

In our study, neem and tulsi possessed adequate smear layer removing capacity and also the push-out bond strength. The findings of our study were in accordance with those of Kale et al. [12].

Herbal irrigants can be used when the patient is allergic to conventionally used endodontic irrigants. Given similar efficiency, herbal irrigants may soon serve as substitutes for chemical alternatives [13].

In vitro results are promising, but preclinical and clinical trials are necessary to confirm their biocompatibility and safety for use as intracanal irrigants and medicaments [14].

Limitations

Lab conditions in the study may limit clinical application as it does not simulate oral conditions accurately. Factors such as blood and tissue remnants in real-world settings may affect irrigant performance. Cleaning curved canals is challenging, while easier needle penetration in wider premolars may impact efficacy, particularly in narrower posterior teeth.

## Conclusions

This study highlights the promising potential of herbal irrigants like neem extract and tulsi leaves with rose water extract in endodontic therapy. These biocompatible alternatives demonstrated adequate smear layer removal and push-out bond strength, indicating their viability as endodontic irrigants. Sodium hypochlorite exhibited the highest efficacy in eliminating the smear layer, while chlorhexidine showcased the strongest push-out bond strength. These findings emphasize the importance of selecting irrigants that efficiently remove the smear layer and enhance the adhesion of root canal filling materials.
